# Probing the Influence of Sulfur–Aromatic Interactions on the Electronic Structure of Gas‐Phase Peptides

**DOI:** 10.1002/chem.202503483

**Published:** 2026-04-02

**Authors:** Laura Pille, Carlos Ortiz‐Mahecha, Bart Oostenrijk, Juliette Leroux, Marion Girod, Konstantin Hirsch, Luke MacAleese, Robert Horst Meißner, Debora Scuderi, Isaak Unger, Vicente Zamudio‐Bayer, Sadia Bari, Lucas Schwob

**Affiliations:** ^1^ Deutsches Elektronen‐Synchrotron DESY Hamburg Germany; ^2^ Institute for Interface Physics and Engineering Hamburg University of Technology Hamburg Germany; ^3^ Department of Physics University of Hamburg Hamburg Germany; ^4^ Université Claude Bernard Lyon 1, ISA UMR 5280, CNRS Villeurbanne France; ^5^ Helmholtz‐Zentrum Berlin für Materialien und Energie Abteilung für Hochempfindliche Röntgenspektroskopie Berlin Germany; ^6^ Institut Lumière Matière (iLM) UMR5306 CNRS & UCBL Lyon France; ^7^ Institute of Surface Science Department of Atomistic Corrosion Informatics, Helmholtz‐Zentrum Hereon Geesthacht Germany; ^8^ Institut de Chimie Physique Université Paris‐Saclay, CNRS UMR8000 Orsay France; ^9^ Department of Physics and Astronomy Uppsala University Uppsala Sweden; ^10^ Zernike Institute for Advanced Materials University of Groningen Groningen Netherlands

**Keywords:** conformational analysis, NEXAMS, NEXAFS, noncovalent interactions, peptides, S⋯π interactions, UVPD, x‐ray action spectroscopy

## Abstract

Non‐covalent sulfur–aromatic (S⋯π) interactions play a crucial role in stabilizing the structure of proteins and have been associated with neurodegenerative diseases. We investigated the influence of the S⋯π interaction on the electronic structure and fragmentation behavior of sequence‐isomer model peptides by means of ultraviolet photodissociation (UVPD) and near‐edge x‐ray absorption mass spectrometry (NEXAMS). The studies revealed distinct fragmentation behavior for one of the model peptides under both valence and core–shell electronic excitation, with characteristic fragmentation channels that serve as potential fingerprints of sulfur–aromatic interactions. Moreover, core–shell excitations at the carbon K‐edge revealed significant shifts between the S⋯π peptide and the control peptides in the aromatic $C1s\to \pi_{C=C}^{*}$ transitions, indicating changes in the electronic structure due to S⋯π interactions. Enhanced sampling molecular dynamics and quantum mechanical calculations reveal the influence of the sulfur orientation, providing insights into the fundamental nature of S⋯π interactions.

## Graphical abstract

Just as fingerprints reveal identity, spectroscopic fingerprints reveal molecular secrets. In this article, we show parts of the unique fingerprint of the sulfur–aromatic interaction. By measuring how this force alters the electronic structure of gas‐phase peptides, we provide a fundamental understanding of interactions that give insights into the stability and properties of biomolecules.

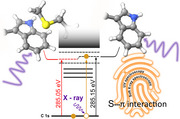

## 1 Introduction

The biological functions of proteins depend on the amino‐acid sequence and the 3D structure, which is stabilized by intramolecular interactions such as van der Waals forces [1]. These noncovalent interactions are critical for protein folding and stability, ultimately determining the functional capacity of proteins. Among these van der Waals bonds, interactions involving sulfur atoms are prevalent in chemical and biological systems [2, 3]. One such noncovalent interaction in proteins is the sulfur–aromatic (S⋯π) interaction, where the sulfur atom of a methionine or cysteine residue interacts with the π‐electron cloud of an aromatic ring of a tyrosine, phenylalanine, or tryptophan residue. Numerous studies and protein database analysis indicated that this interaction contributes to stabilizing the structure of at least one‐third of all known proteins [4, 5]. This interaction was first identified in the late 1970s by Morgan and coworkers, who demonstrated that the divalent sulfur atom could engage in favorable interactions with aromatic systems [6]. The sulfur aromatic interaction is driven by the highly polarizable sulfur with its open 3p and empty 3d orbitals, which facilitate the interaction with aromatic π electrons [4, 7]. The bond energy of this interaction ranges from 1.0 to 2.0 kcal/mol (0.04–0.09 eV), comparable to that of an ionic salt bridge [8, 9]. Further studies have shown that the sulfur–aromatic interaction is most prominent at distances between 5 and 6 Å, although the exact orientation of the sulfur atom relative to the aromatic ring is still a subject of ongoing research [10]. Beyond their role in protein stabilization, it has been suggested that by formations of multicenter three‐electron bonds, the sulfur–aromatic interactions may also be involved in electron‐hole transport within proteins [11]. This highlights the biological relevance of the sulfur–aromatic interaction, which is further emphasized by its involvement in various pathologies. Neurodegenerative diseases such as Alzheimer's and Creutzfeldt–Jakob disease, for example, have been linked to the oxidative modification of methionine residues [5, 12]. Oxidation of the sulfur atom can disrupt the S⋯π interactions, leading to conformational changes in proteins and ultimately a loss of function [13]. Moreover, the sulfur–aromatic interaction is gaining increasing attention in the fields of drug discovery and catalysis [4]. Understanding the nature and strength of the interaction is essential for the development of targeted therapeutic strategies and innovative organo‐catalysts. Therefore, a comprehensive understanding of the sulfur–aromatic interaction is crucial for advancing our knowledge of protein structures and the implications of these interactions in biological processes.

Studying these noncovalent interactions within biomolecular ions in the gas phase guarantees the absence of solvent effects and other environmental influences, such as those present in condensed or liquid phase environments. Electrospray ionization (ESI) with tandem mass spectrometry (MS) has proven to be a powerful tool for the generation and analysis of gas‐phase biomolecular ions, enabling the investigation of protein structures [14]. Photodissociation techniques can complement the structural information obtained from ESI‐MS. For example, ultraviolet photodissociation (UVPD) enables selective valence excitation to induce fragmentation, which provides insights into the molecular structure [15, 16, 17]. Near‐edge x‐ray absorption mass spectrometry (NEXAMS) extends this approach by targeting core atomic levels, offering element‐specific activation and deeper insights into the electronic structure of molecular systems. These mass spectrometry‐based action spectroscopy techniques have been of growing interest in the past decade. NEXAMS in particular has been successfully used to identify photodissociation processes and structural features of peptides and proteins [18, 19, 20, 21, 22, 23]. Furthermore, previous studies have shown that noncovalent interactions, such as hydrogen bonds, can lead to characteristic signatures in x‐ray absorption spectra [24, 25, 26, 27].

Motivated by these findings, the present study aims at identifying a spectroscopic fingerprint associated with sulfur–aromatic (S⋯π) interactions. We applied UVPD and NEXAMS to investigate the influence of this noncovalent interaction on the electronic and geometric structure of biomolecular ions by probing the $\pi \pi^{*}$ excitations and core‐level resonances at the carbon K‐edge, targeting aromatic groups. The influence of S⋯π interactions on the electronic structure of peptides in the gas phase is investigated using custom peptides as synthetic models, in which the noncovalent interaction can be investigated in isolation from the complexity of larger biomolecules. The peptides consist of 10 amino‐acid residues and were designed to systematically vary the 1D distance in the peptide sequence between the aromatic ring of tryptophan (Trp, W) and the divalent sulfur of methionine (Met, M). However, due to the flexibility of the peptide backbone and side‐chains, this 1D sequence distance does not necessarily correlate with the 3D spatial distance between the residues. The choice of tryptophan was based on protein structural analyses, which indicate a preference for methionine to reside near tryptophan or tyrosine [28, 29]. To ensure a fixed positive charge at the N‐terminus and potentially enhance the S⋯π interaction [5, 30], arginine (Arg, R) was included in the peptide sequence. Finally, glycine (Gly, G) residues were added to create the desired distances between the aromatic ring and the divalent sulfur, enabling a systematic study of the sulfur–aromatic interaction with the four peptide samples given in Table 1.

**TABLE 1:** Peptide sequence of all studied samples.

Peptide sequence	Label
Arg‐**Trp**‐**Met**‐Gly‐Gly‐Gly‐Gly‐Gly‐Gly‐Gly	RWMG_7_
Arg‐**Trp**‐Gly‐Gly‐**Met**‐Gly‐Gly‐Gly‐Gly‐Gly	RWG_2_MG_5_
Arg‐**Trp**‐Gly‐Gly‐Gly‐Gly‐**Met**‐Gly‐Gly‐Gly	RWG_4_MG_3_
Arg‐**Trp**‐Gly‐Gly‐Gly‐Gly‐Gly‐Gly‐**Met**‐Gly	RWG_6_MG

## 2 Results and Discussion

### 2.1 Ultraviolet Photodissociation (UVPD)

A potential sulfur–aromatic interaction in our four model peptides was examined using UVPD at 266 nm to excite the aromatic amino acid, tryptophan [31]. This wavelength selectively excites the highest occupied molecular orbital (HOMO)–lowest unoccupied molecular orbital (LUMO) $\pi \pi^{*}$ transition of the aromatic side chain, allowing us to probe potential local interactions [32]. Details regarding the experimental method are provided in the Experimental Section, and information on specific data treatment is available in the Supporting Information (SI).

**FIGURE 1:**
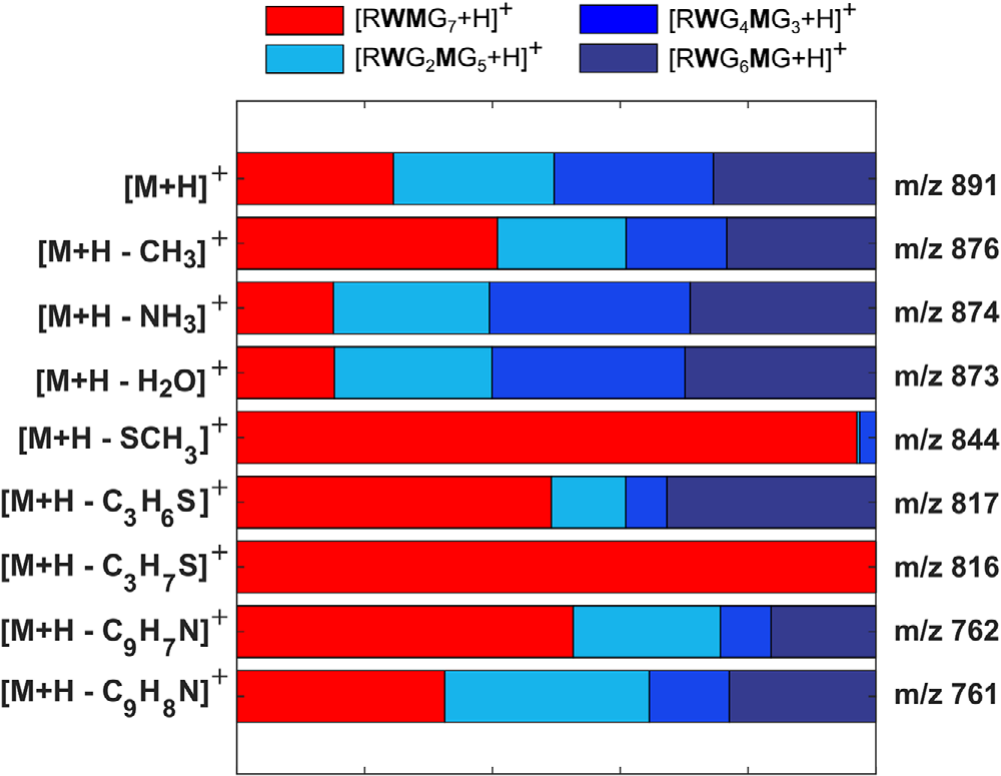
Relative branching ratios of selected fragments of four model peptides, measured at 266 nm. For each fragment, intensities are summed across all samples and then used to normalize (see Supporting Information for more information).

The analysis of the obtained mass spectra (see Figure S1), presented in the stacked bar plot in Figure 1, reveals distinct fragmentation patterns between the peptides. While the neutral losses of ${\mathrm{NH}}_{3}$ and $H_{2}O$ exhibit similar intensities across all four samples, the losses of sulfur‐containing fragments show more pronounced variations. Notably, the [${\mathrm{RWMG}}_{7}$+H]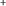 sample, in red, exhibits a unique fragmentation pattern characterized by the prominent loss of ${\mathrm{SCH}}_{3}$ and $C_{3}$ $H_{7}S$, both originating from the methionine residue. Moreover, the ${\mathrm{CH}}_{3}$ loss is slightly enhanced in the spectrum of [${\mathrm{RWMG}}_{7}$+H]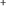. These three specific fragment losses are either absent or present at lower intensities in the spectra of the three other samples, where the spacing between the Trp and Met residues in the amino—acid sequence is larger, suggesting a specific dissociation processes for [${\mathrm{RWMG}}_{7}$+H]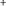. A similar behavior has been reported by Talbert et al., who performed UVPD spectroscopy on α ‐ helical peptides containing aromatic and sulfuric residues [33]. The authors have observed ${\mathrm{CH}}_{3}$, ${\mathrm{SCH}}_{3}$ and $C_{3}$ $H_{7}S$ losses from methionine after excitation in the aromatic group, only when both residues were in close spatial configuration, that is, one loop away in the helix. They proposed that an efficient energy transfer mechanism occurs from the aromatic to the sulfuric residue following $\pi \pi^{*}$ excitation, leading to specific C–S bond cleavages. Correspondingly, loss of ${\mathrm{SCH}}_{3}$ and ${\mathrm{CH}}_{3}$ is attributed as the dominant fragmentation pathway, whereas C–C bond cleavages within the Met side chain are considered as secondary fragmentation paths. The specific photodissociation pathways observed in the [${\mathrm{RWMG}}_{7}$+H]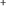 peptide can be interpreted in the same manner. Upon the absorption of a 266 nm photon by the aromatic residue, energy is transferred to the Met residue, where the sulfur atom acts as an acceptor, thus enabling cleavage within the methionine side chain. The mass spectra (see Figure S1) support the proposed mechanism, as the absolute intensities for the loss of ${\mathrm{SCH}}_{3}$ and ${\mathrm{CH}}_{3}$ are significantly higher than those for the loss of $C_{3}$ $H_{7}S$, indicating that C─S bond cleavage is the more dominant process. This process almost only appears to be possible in the [${\mathrm{RWMG}}_{7}$+H]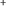 peptide, which suggests that the spatial proximity of the Trp and Met residues is favorable in the [${\mathrm{RWMG}}_{7}$+H]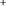 peptide. We hypothesize that this characteristic fragmentation pattern can be used as an indicator for an existing S⋯π interaction for the [${\mathrm{RWMG}}_{7}$+H]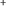 peptide. To provide more detailed information about the properties of this interaction and its impact on the electronic structure of peptides, we conducted a comprehensive study using NEXAMS.

### 2.2 Near‐Edge X‐Ray Absorption Mass Spectrometry (NEXAMS)

**FIGURE 2:**
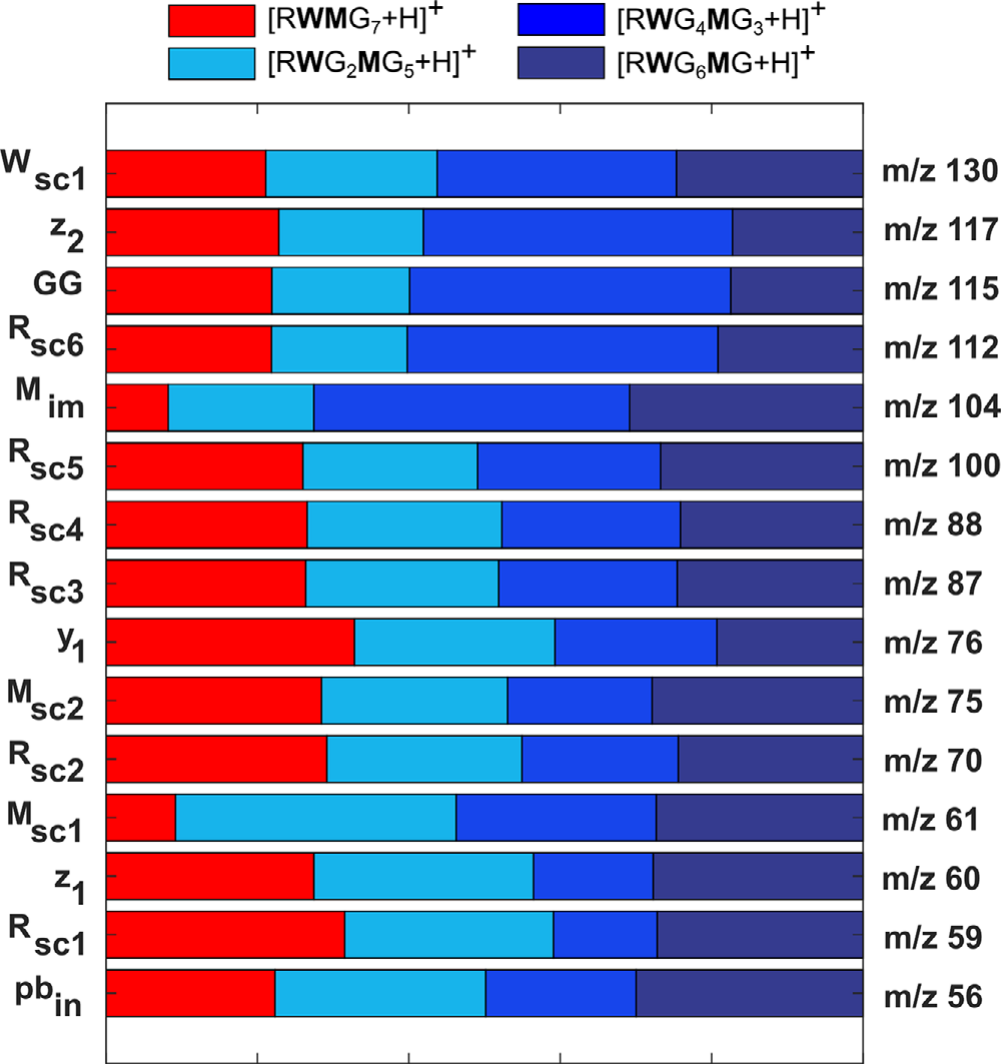
Relative branching ratios for all fragments of four model peptides measured at 288 eV. For each fragment, intensities are summed across all samples and then used to normalize (see SI for more information). sc, side‐chain fragment; im: immonium ion.

**FIGURE 3:**
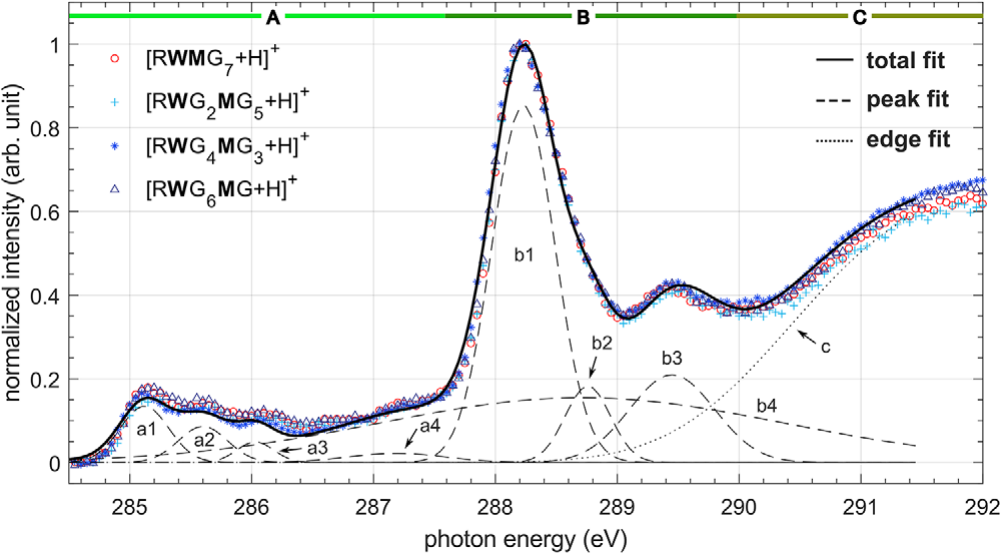
Experimental carbon K‐edge total ion yield spectra of [${\mathrm{RWMG}}_{7}$+H]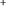, [${\mathrm{RWG}}_{2}{\mathrm{MG}}_{5}$+H]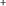, [${\mathrm{RWG}}_{4}{\mathrm{MG}}_{3}$+H]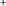 and [${\mathrm{RWG}}_{6}\mathrm{MG}$+H]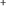 and the fitted Gaussian peaks performed for [${\mathrm{RWG}}_{4}{\mathrm{MG}}_{3}$+H]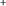. The experimental spectra are normalized to the maximum intensity of the C 1s $\to \pi_{CONH}^{*}$ resonance (peak b1, 288.20 eV).

We explored the influence of sulfur–aromatic interactions on the electronic properties of our model peptides by probing resonant absorptions at the carbon K‐edge. Figure 2 presents the fragmentation patterns of all four peptides acquired at an excitation energy of 288 eV. A detailed description of the experimental technique is provided in the Experimental Section. Information regarding data treatment, the mass spectra (Figure S2), and the list of identified fragments (Table S1) is provided in the SI. Overall, the fragmentation pattern of all four samples is comparable. It can be observed that the mass spectra are dominated by fragments originating from the arginine side chain, including ions at *m/z* 70, 88, 87, and 112. The fragment ion at *m/z* 130 is attributed to the dissociation of the tryptophan side chain W_sc1_. In addition to the Arg and Trp fragments, the immonium ion of methionine (*m/z* 104) and its side chain fragments M_sc1_, and M_sc2_ (*m/z* 61 and 75) were identified. Noticeable is that the [${\mathrm{RWMG}}_{7}$+H]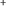 peptide shows a lower intensity of Met‐related fragments, M_im_ and M_sc1_. In addition to fragments originating from specific amino‐acid residues, we observed fragments resulting from peptide bond cleavages, such as pb_in_, z_1_, y_1_, GG, and z_2_ ions (*m/z* 56, 60, 76, 115 and 117). While we refer to standard peptide fragmentation nomenclature [34, 35], including a_n_, b_n_, and c_n_ ions from the N‐terminus and x_n_, y_n_, and z_n_ ions from the C‐terminus, some fragment assignments do not follow these conventions. These include side‐chain‐specific ions as well as internal fragments, which are defined in detail in the Table S1 provided in the SI. The total ion yield for each peptide was determined by summing the yields of all detected fragment ions presented in Figure 2 at each photon energy. These total ion yield spectra are shown in Figure 3 for all four peptides and three distinct regions, denoted in the upper part of Figure 3, could be identified: A (284.8–287.5 eV), B (287.6–290 eV), and C (>290 eV). Region A in the C K‐edge spectrum is characteristic of C 1s $\to \pi^{*}$ excitations in aromatic residues. [18, 36] This region can be further sliced into four distinct peaks, aligning with previous studies on Trp [37, 38]. These peaks correspond to specific electronic transitions within the Trp residue. The first peak at 285.12 eV (a1) corresponds to the excitation from carbons of the benzene ring of tryptophan's indole to the $\pi^{*}e_{2u}$ orbital [39]. The following two peaks, at 285.60 eV (a2) and 286.02 eV (a3), are primarily attributed to excitations involving carbon atoms within the pyrrole ring of Trp indole. Specifically, the a2 peak is mainly associated with transitions involving C = C carbons, while the a3 peak corresponds to transitions involving C–N carbons, as discussed in previous studies [40, 41]. The broad shoulder observed between 286.50 and 287.60 eV (a4) likely results from overlapping transitions, potentially involving all residues, including C 1s $\to \sigma_{CH}^{*}/\pi^{*}$ [38], C 1s $\to \pi_{C=O}^{*}$, C 1s $\to \sigma_{CHS}^{*}$ [42], as well as contributions from Rydberg states. In region B, the most intense peak at 288.20 eV (b1) arises from C 1s $\to \pi_{CONH}^{*}$ transitions in the peptide backbone [43]. This dominance can be attributed to the relatively large size of the 10‐amino‐acid peptides, which contain a higher abundance of peptide‐bond functional groups compared to the amount of Trp, Met, and Arg residues. The subsequent peak at approx. 288.70 eV (b2) stems from transitions involving arginine residues, as indicated by previous studies on arginine [44]. The resonance at 289.45 eV (b3) can be assigned to the C 1s $\to \sigma_{CC}^{*}$ transition in glycine moieties, consistent with the findings of Plekan et al. [42]. In region C, we added a step function (c) to the fit of the spectra to account for the C 1s ionization edge of the model peptides at approximately 290.20 eV.

The analysis of the total ion yield spectra shows features originating from electronic transitions in all four types of residue present in the peptides. Although the peptide sequence contains a majority of Gly residues, the total ion yield spectra still exhibit distinct features associated with individual residues such as Trp, Met, and Arg. This is in alignment with previous studies showing that even small spectral contributions from individual residues can be identified in the overall ion yield spectrum [45], underscoring the sensitivity of the technique to specific residue characteristics. While comparing the total ion yield spectra across all four samples, the qualitative resemblance of the spectra indicates a high degree of structural similarity between the peptides despite being sequence‐isomers. Therefore, a more sensitive approach is required to distinguish potential differences between the isomers and ultimately provide a spectroscopic fingerprint of a sulfur–aromatic interaction at the carbon K‐edge. To that end, we analyzed the partial ion yield spectra of various fragments.

**FIGURE 4:**
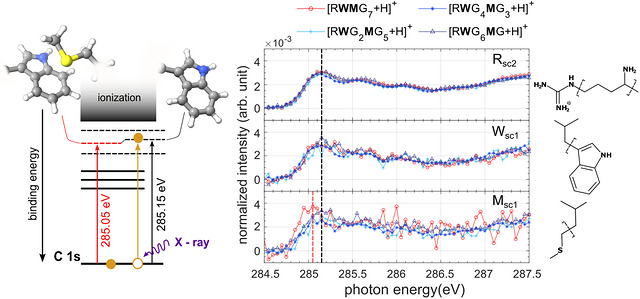
Left: Energy level diagram for aromatic carbon $1s\to \pi^{*}$ photoexcitation. The diagram focuses on changes in the $\pi^{*}$ orbital, while potential alterations of the occupied (HOMO) orbitals are not taken into account (solid horizontal lines: occupied states; dashed horizontal lines: unoccupied states; not to scale). Right: Partial ion yield for R_sc2_ (*m/z* 70, $C_{4}$ $H_{8}N$ ^+^), W_sc1_ (*m/z* 130, $C_{9}$ $H_{8}$ $N^{+}$), and M_sc1_(*m/z* 61, $C_{2}$ $H_{5}$ $S^{+}$) fragments. Black vertical line at 285.15 eV highlights the excitation from carbons of the benzene ring of Trp to the $\pi^{*}$ orbital and red vertical line at 285.05 eV highlights the energy redshift of the C 1s $\to \pi_{C=C}^{*}$ resonance in the case of the [${\mathrm{RWMG}}_{7}$+H]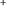 peptide observed in the M_sc1_ fragment.

By analyzing the partial ion yield spectra for side‐chain fragments of arginine (R_sc2_, *m/z* 70), tryptophan (W_sc1_, *m/z* 130), and methionine (M_sc1_, *m/z* 61), we aimed at gaining deeper insights into the local environment of specific amino‐acid residues. To focus on the region characteristic of aromatic residues (region A in the C K‐edge spectrum, see Figure 3), Figure 4 shows the partial ion yield in the range between 284.5 and 287.5 eV. While the partial ion yield spectra for the Arg and Trp fragments show no significant differences compared to the total ion yield across all four samples, the M_sc1_ fragment of [${\mathrm{RWMG}}_{7}$+H]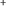 exhibits a significant energy redshift of ∼100 meV in the C 1s $\to \pi_{C=C}^{*}$ resonance related to the benzene moiety of the tryptophan side chain, from 285.15 eV (black vertical dashed line) to 285.05 eV (red vertical dashed line). In contrast, the other three samples show the transition for R_sc1_, W_sc_, and M_sc1_ centered at 285.15 eV. This suggests that the [${\mathrm{RWMG}}_{7}$+H]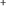 peptide creates a unique local environment in which the sulfur‐containing methionine side chain perturbs the electronic structure of the tryptophan residue (see Figure 4, left schematic). Although the C 1s $\to \pi_{C=C}^{*}$ transitions are localized on Trp, the resulting redshift is only observed in a methionine fragment. We propose that this may indicate that the specific perturbation of the electronic structure due to the S⋯π interaction is maintained up to the point of fragmentation, allowing it to be observed in the resulting Met fragment. Additional insight into the suggested interpretation about the observed phenomenon is provided in the theoretical section.

In addition to the structural insights gained from the x‐ray absorption spectra, the quantitative analysis of the mass spectra provides further information on the fragmentation processes. It has been shown that following photoexcitation and Auger–Meitner decay, electronically excited peptide cations predominantly undergo internal conversion followed by intramolecular vibrational energy redistribution, leading to statistical fragmentation [21, 46]. As mentioned above, we observe a high number of fragments originating from the arginine side chains (see Figure 2), which can be attributed to the high basicity of the arginine side chain. The high proton affinity of the guanidino group and the stabilization resulting from the delocalized electron density leads to a strong binding of the proton to the arginine side chain. As demonstrated by Dongre et al. [47] in the context of collision‐induced dissociation (CID), this fixed charge results in a higher energy barrier for fragmentation. It is worth noting, however, that the high energy deposited upon x‐ray absorption can be enough to overcome this energy barrier [46]. Nevertheless, peptide bond cleavages in the frame of the “mobile‐proton” model [48] are less favorable, and protonated arginine‐related fragments are more likely to form. Indeed, the overall fragmentation pattern observed in our study is consistent with the expected influence of fixed charge in CID experiments, as most of the observed fragments can also be found within CID experiments [49].

**FIGURE 5:**
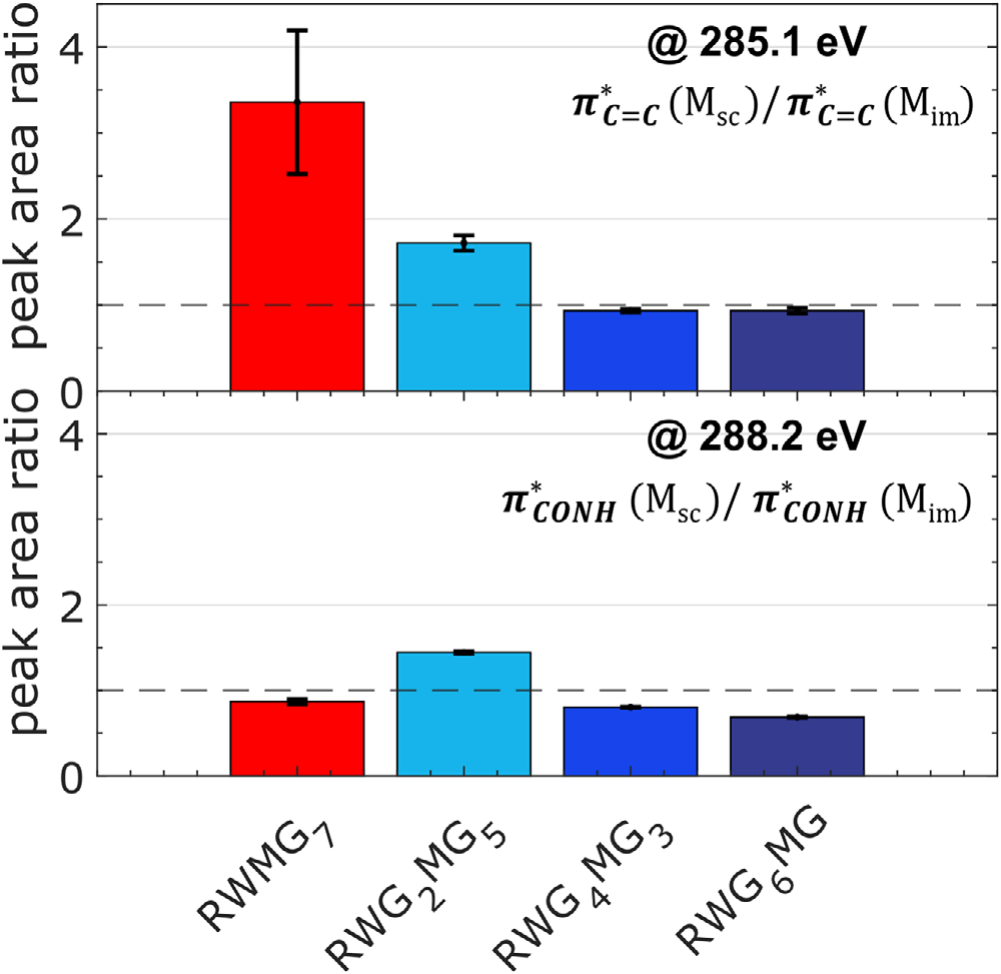
Influence of the sulfur–aromatic interaction on the fragmentation pattern. Ratios between normalized partial ion yields of M_sc1_ and M_im_ for all samples. Top: measured upon C 1s $\to \pi_{C=C}^{*}$ excitation (285.1 eV) and bottom: C 1s $\to \pi_{CONH}^{*}$ excitation (288.2 eV).

Nevertheless, as discussed in the UVPD section, a specific fragmentation pattern was observed for [${\mathrm{RWMG}}_{7}$+H]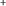 upon valence excitation of the aromatic system. Extending this analysis to the x‐ray data, we aimed to identify potential variations in the fragmentation behavior associated with core–shell excitation of carbon atoms within the aromatic system. A comparative analysis of the methionine‐related side‐chain fragment, M_sc1_, and the immonium ion, M_im_, is a useful indicator for understanding the fragmentation patterns of our peptide samples. Indeed, the fragmentation processes leading to the formation of the side‐chain fragments may not exhibit the same statistical distribution as those observed in the formation of the immonium ion that is formed through cleavages of the CO‐NH and $C_{\alpha }$‐CO bonds of the peptide backbone at the methionine residue. As discussed earlier, the fixed charge increases the likelihood of statistically favored Arg fragments. In our x‐ray absorption experiment, the stable Met immonium ion is consistently observed, regardless of where the photon is absorbed by the system. In contrast, side‐chain fragments often originate from more direct and specific bond cleavages within the side chain of the amino acids [36, 50]. To identify the behavior of the side chain and the immonium ion of Met, we show in Figure 5, the fragmentation ratio of M_sc1_ / M_im_ upon a) C 1$s\to \pi_{C=C}^{*}$ excitation (285.1 eV) and b) C 1s $\to \pi_{CONH}^{*}$ excitation (288.2 eV). At 288.2 eV, the fragment ratios between sequence‐isomers are approximately 1, indicating comparable formation of Met side‐chain and immonium ion fragments across all four peptides. This consistent behavior is expected, as the excitation of the peptide bonds leads to energy redistribution throughout the entire peptide, enabling the formation of both M_sc_ and M_im_ fragments. However, upon $C1s\to \pi_{C=C}^{*}$ excitation, a significant increase in the M_sc1_ /M_im_ ratio is observed for [${\mathrm{RWMG}}_{7}$+H]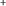. This indicates a preferential formation of methionine side‐chain fragments over the methionine immonium ion when core excitation occurs at the aromatic group. This high M_sc1_ /M_im_ ratio observed for the C 1s $\to \pi_{C=C}^{*}$ transition is essentially due to the very low yield of the M_im_ fragment, which suggests that, in addition to the observed M_sc1_ fragment, smaller methionine side‐chain fragments are produced (e.g., ${\mathrm{SCH}}_{3}$, S, ${\mathrm{CH}}_{3}$...) but not measured as they fall outside the accessible *m/z* window in these measurements. This observed trend is similar to our previous findings on methionine enkephalin (MetEnk) [36]. The comparative analysis of the fragment ratio between MetEnk and leucine enkephalin (LeuEnk) revealed an increase in the relative yield of methionine‐related side chain fragments (*m/z* 75), coupled with a decrease of M_im_ ions upon C 1s $\to \pi_{C=C}^{*}$ excitation in the aromatic rings (phenylalanine and tyrosine) of the peptide. The [${\mathrm{RWMG}}_{7}$+H]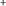 peptide exhibits a strikingly similar trend (higher M_sc1_ /M_im_ ratio), supporting the hypothesis that the fragmentation process upon C 1$s\to \pi_{C=C}^{*}$ excitation does not mainly involve global charge or energy migration along the peptide backbone. Instead, it proceeds via a direct charge or an energy transfer from the tryptophan side chain to the methionine side chain, leading to the production of the M_sc1_ (and smaller side—chain fragments). This process is likely favored in conformers where the two chemical groups are in close spatial proximity, stabilized by a noncovalent sulfur–aromatic interaction.

We have demonstrated that the [${\mathrm{RWMG}}_{7}$+H]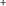 peptide exhibits distinct dissociation behavior upon both UV and x‐ray absorption. Our results indicate that the close spatial proximity of methionine and tryptophan influences the fragmentation pattern and can result in a shift in the C 1s $\to \pi_{C=C}^{*}$ transition observable in specific fragments. These findings suggest that [${\mathrm{RWMG}}_{7}$+H]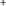 can serve as a model system for studying sulfur–aromatic interactions, as evidenced by its unique behavior under both valence and core–shell excitation. We have now identified a distinct spectroscopic fingerprint of the sulfur–aromatic interaction under gas‐phase conditions. This highlights the capability of NEXAMS to sensitively probe such interactions in isolated biomolecules. The observed fragmentation behavior and electronic shifts serve as indicators of the noncovalent S⋯π interaction.

**FIGURE 6:**
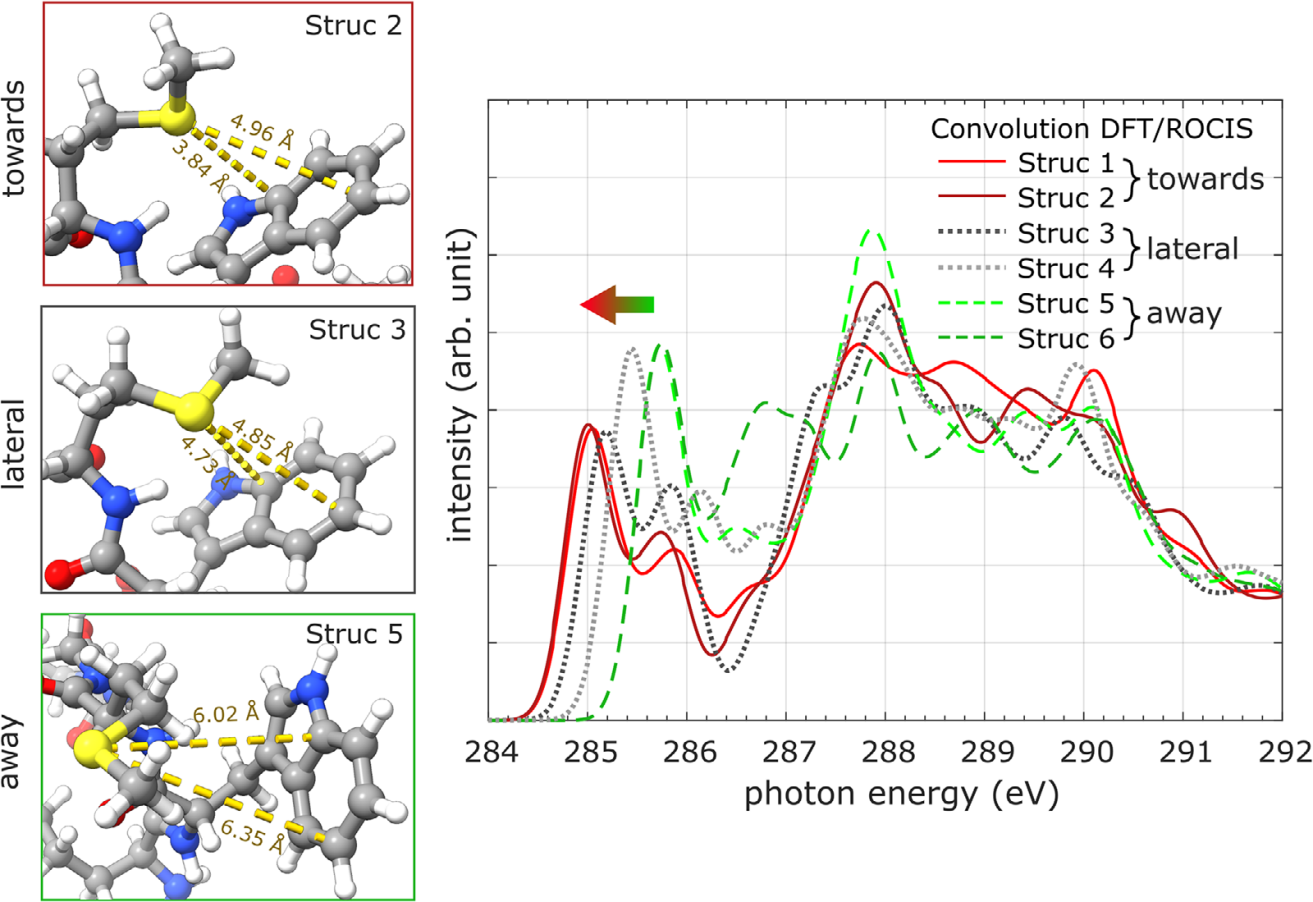
Left: Zoom in the methionine and tryptophan spatial arrangement of three example conformations of [${\mathrm{RWMG}}_{7}$+H]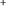, selected from REMD‐sampled dominant populations, Struc. 2 (red), Struc. 3 (gray), and Struc. 5 (green). Right: Convolution of the DFT/ROCIS x‐ray absorption lines for six structures, with the lone pair pointing toward the ring (red), away from the ring (green), and intermediate lateral orientation (gray). The convolutions were broadened using a pseudo‐Voigt profile with a 0.5 eV FWHM and shifted by 11.4 eV to align the peptide bond resonance near 288 eV.

To further support and extend the experimental observation, replica exchange molecular dynamics (REMD) simulations [51] were performed to identify representative structures of [${\mathrm{RWMG}}_{7}$+H]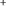, analyzing the spatial proximity between the sulfur (Met) and aromatic ring (Trp). A detailed description of the theoretical methods is provided in the SI. Potential energy surface analysis identified the most populated [${\mathrm{RWMG}}_{7}$+H]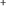 conformations as those with a sulfur–aromatic distance ranging from 3.8 to 7.7 Å (see Figure S4). Therefore, the identified S⋯π interaction motifs are based on geometric criteria, specifically the distance between the Met sulfur atom and the Trp aromatic ring, similarly to previous PDB analyses [5, 13]. We focused on structures exhibiting different orientations of the sulfur lone pair relative to the aromatic ring, identifying two distinct extremes: oriented toward the ring and away from the ring (see Figure 6). As shown in Figure 6, the C 1s $\to \pi_{C=C}^{*}$ transition exhibits a shift depending on the sulfur orientation: a redshift of up to 800 meV in the C 1s $\to \pi_{C=C}^{*}$ transitions is observed when the sulfur lone pair moves from the orientation away (green structure and dashed line) to toward the aromatic ring (red structures and plain lines), while transitions associated with the peptide backbone (around 288 eV) remain mostly unaffected. Moreover, we could identify conformer structures with intermediate sulfur orientations that cannot be clearly classified as pointing toward or away from the aromatic ring (see Figure S5 in the SI for the intermediate structure). In Figure 6, Struc. 3 and 4 (gray dotted lines) represent calculated spectra for structures with more lateral orientation but still in the distance range of 4.7–5 Å, respectively. Both structures follow the trend of the redshift. The trend indicates that the proximity of the methionine residue near the tryptophan side chain significantly influences the electronic transitions within the aromatic group. This is consistent with the experimentally observed redshift of the C 1s $\to \pi_{C=C}^{*}$ transitions. Similar studies have shown a spatial dependence of the electronic transitions in π–π stacking interactions, where the distance between aromatic side chains was found to influence intermolecular resonant energy transfer [52, 53, 54]. Extending this idea to sulfur–aromatic interactions, our results indicate that within the S⋯π interaction, the orientation of the sulfur atom with its lone pair directed toward the aromatic ring (see Figure 6 Struc. 1 and 2, red lines) has a more pronounced redshift over geometries orienting the lone pair lateral (Struc. 3 and 4, gray dotted lines) or away from the ring (Struc. 5 and 6, green dashed lines). To better understand the origin of the observed redshift, we performed an atomic contribution electronic transition density analysis [54] in the calculated x‐ray absorption spectra for each structure in the first intense peak (between 284.9 and 285.8 eV). These transitions are solely assigned to C 1s $\to \pi_{C=C}^{*}$ excitations, primarily involving carbon atoms of the benzene ring of the Trp side chain. However, in the analysis of the C 1s $\to \pi_{C=C}^{*}$ transitions, the atomic contribution of sulfur as an acceptor is not observed, since internal conversion by a nonradiative spin–vibronic mechanism is not considered [55]. The specific charge transfer carbon and sulfur is expected to be quantified in the weaker C 1$s\to \sigma_{CHS}^{*}$ transitions above 287.1 eV combined with the Rydberg states [42]. Nevertheless, sulfur–aromatic interactions, as well as contributions from other atoms, can still perturb the electronic C 1s $\to \pi^{*}C=C$ transitions in the first excited states and induce the redshift via, for example, orbital hybridization.

Although the exact mechanism by which the S⋯π interaction affects the C 1s $\to \pi_{C=C}^{*}$ transitions remains unclear, a potential mechanism is that the sulfur–aromatic interaction perturbs the energy levels of the aromatic side chain by altering orbital hybridization, changing the energy level of the excited state of the Trp residue ($\pi^{*}$ orbital) and thereby reducing the energy of the electronic transitions (see Figure 4, left schematic). More detailed information on excited‐state charge transfer involving sulfur–carbon orbital coupling could be observed by analyzing sulfur L‐ and K‐edge absorption spectra, including spin‐orbit coupling effects. Further experimental studies, such as UV spectroscopy across the $\pi \pi^{*}$ transition of the aromatic group, could infer whether the HOMO–LUMO gap exhibits changes, and measurement of the valence photoionization threshold would allow the accurate measurement of the HOMO binding energy and thus assess any changes in the HOMO energy level induced by the S⋯π interaction.

## 3 Conclusion

Through our study of the S⋯π interaction in protonated model peptides, ([${\mathrm{RWMG}}_{7}$+H]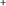 has proven to be an excellent system for studying the sulfur–aromatic interaction. It exhibits a unique mass spectral signature upon photoabsorption in both the UV and soft x‐ray regions. In particular, we have shown the impact of the S⋯π interaction on the electronic structure, and our results demonstrate that the S⋯π interaction significantly alters the electronic structure of the aromatic side chain, which is highlighted by the redshift observed in the C 1$s\to \pi_{C=C}^{*}$ transitions at the carbon K‐edge. These observations provide valuable information about the geometrical arrangement and the resulting changes in electronic structure associated with the S⋯π interaction. Thus, we identified a distinct spectroscopic fingerprint that serves as an indicator of the S⋯π interaction. In addition, our theoretical simulations not only support the experimental findings but also propose that the orientation of the sulfur atom with the lone pair directed toward the aromatic ring has a more pronounced effect. Hence, we could show that our combined experimental and theoretical approach proves to be sensitive to minor changes induced by intramolecular noncovalent interactions, allowing us to extract insights about conformational motifs and their electronic structure. The detailed pathways by which this interaction alters the peptide electronic structure continue to be studied. Further theoretical and experimental investigations are required to fully elucidate the nature and strength of this interaction and its impact on the spectroscopic properties of peptides containing sulfur–aromatic motifs.

## 4 Experimental Section

All peptide samples with sequences ${\mathrm{RWMG}}_{7}$, ${\mathrm{RWG}}_{2}$ ${\mathrm{MG}}_{5}$, ${\mathrm{RWG}}_{4}$ ${\mathrm{MG}}_{3}$, and ${\mathrm{RWG}}_{6}\mathrm{MG}$, used in this study were synthesized by ProteoGenix (Schiltigheim, France), with a purity of 95%, and were used without further purification. Sample solutions were prepared at a concentration of 30 μM in a 1:1 mixture of water and methanol.

### 4.1 Ultraviolet Photodissociation (UVPD)

The experiments were conducted using a modified hybrid quadrupole‐orbitrap Q‐Exactive mass spectrometer (Thermo Fisher Scientific, San Jose, CA, USA) equipped with a HESI ion source. The instrument was further modified by fitting a silica window on the rear of the higher‐energy collisional dissociation (HCD) cell to enable laser irradiation of trapped ions [56, 57]. Electrospray ionization (ESI) was performed in positive ion mode with a spray voltage of 4500 V and a flow rate of 5 μL/min to introduce the synthesized samples. The sheath gas and auxiliary gas (nitrogen) flow rates were set to 20 and 15 (arbitrary units), respectively, with a HESI vaporizer temperature of 250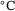. The ion transfer capillary temperature was also set to 250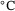. The S‐lens radio‐frequency (RF) level was set to 55 (arbitrary units). The orbitrap resolution was set to 140 000. The automatic gain control (AGC) target was 5×10^6^, and the maximum injection time was set to 100 ms. An *m/z* window of 3.0 Th was applied for precursor isolation. A BrillantB Nd:YAG laser (Quantel, Les Ulis, France) was coupled to this setup to perform UVPD of the ions inside the mass spectrometer. The 4th harmonic at λ = 266 nm was used with a repetition rate of 20 Hz. The laser beam was cleaned using lenses and diaphragms and was injected into the HCD cell using two dichroic mirrors. The laser energy irradiating the ions was 5 mJ per pulse. For UVPD, the HCD parameters were optimized to prevent CID, with the collision energy set to 3 eV and irradiation times to 1000 ms, depending on the experiment.

### 4.2 Near‐Edge X‐ray Absorption Mass Spectrometry (NEXAMS)

The experiments were carried out at the UE52 PGM Ion Trap beamline of the BESSY II synchrotron (HZB Berlin, Germany) [58]. Electrospray ionization (ESI) was employed in positive ion mode to introduce the synthesized samples into the gas phase. The ions were then focused into a beam using a radio‐frequency (RF) ion funnel and guided through an RF hexapole ion guide to a quadrupole mass filter, which selectively filtered the ions based on their mass‐to‐charge ratio (*m/z*). Following mass selection, the ions passed through a 90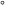 bender to prevent neutral molecules from entering the interaction region. The selected ions were accumulated and stored in a cryogenic linear RF ion trap, where they were cooled using helium buffer gas. The trap temperature was maintained between 10–20 K during the experiments. Once the ions were accumulated, they were irradiated with soft x‐ray photons for 100 ms, co‐linearly to the RF trap axis. A specific photon energy range was measured by scanning the carbon K‐edge, spanning from 283 to 292 eV with a bandwidth of 100 meV, using slits set to 200 μm and a step size of 50 meV. A photodiode placed downstream of the linear ion trap was used to record the photon flux. The resulting cationic products from photon absorption were analyzed using a reflectron time‐of‐flight mass spectrometer. Due to the instrument design, the detection of photoproducts was limited to a small *m/z* window of 50–150, a range that was chosen because it encompassed the most intense fragments.

## Conflicts of Interest

The authors declare no conflicts of interest.

## Supporting information


**Supporting File**: chem70864‐sup‐0001‐SuppMat.pdf.

## Data Availability

All data supporting the findings of this study are available within the article and its Supporting information (SI). The SI contains computational methods, optimized structures, and mass spectra. Additional data related to this work can be requested from the authors.
