# Supplementary material for: Probing the Influence of Sulfur–Aromatic Interactions on the Electronic Structure of Gas‐Phase Peptides
Source: Chemistry. 2026 Apr 2;32(23):e03483. doi: 10.1002/chem.202503483 (PMC13282918; doi:10.1002/chem.202503483)
Supplement: Supplementary file 1 — Supporting File: chem70864‐sup‐0001‐SuppMat.pdf. [file CHEM-32-e03483-s001.pdf]

Supporting Information

Probing the Influence of Sulfur–Aromatic Interactions on the Electronic Structure of Gas-Phase Peptides

Laura Pille,[a] Carlos Ortiz-Mahecha,[b] Bart Oostenrijk,[a] Marion Girod,[c] Konstantin Hirsch,[d] Juliette Leroux,[a,e] Luke MacAleese,[f] Robert Meißner,[b] Debora Scuderi,[g] Isaak Unger,[h] Vincente Zamudio-Bayer,[d] Sadia Bari,*,[a,i] Lucas Schwob*,[a]

[a] Deutsches Elektronen-Synchrotron DESY, 22603 Hamburg, Germany.
E-mail: sadia.bari@desy.de, lucas.schwob@desy.de

[b] Technical University Hamburg (TU Hamburg), Hamburg, Germany.

[c] Université Claude Bernard Lyon 1, ISA UMR 5280, CNRS, 69100 Villeurbanne, France.

[d] Helmholtz-Zentrum Berlin für Materialien und Energie, 12489 Berlin, Germany.

[e] University of Hamburg, 22607 Hamburg, Germany.

[f] Institut Lumière Matière (iLM), UMR5306 CNRS & UCBL, Lyon, France.

[g] Université Paris-Saclay, CNRS, Institut de Chimie Physique, UMR8000, 91405 Orsay, France.

[h] Uppsala University, 75120 Uppsala, Sweden.

[i] Zernike Institute for Advanced Materials, University of Groningen, 9747 AG Groningen, The Netherlands.

Contents

1 Experimental Section **2**

1.1 Ultraviolet Photodissociation (UVPD) 2

1.2 Near-Edge X-ray Absorption Mass Spectrometry (NEXAMS) 3

2 Computational Methods **6**

1 Experimental Section

1.1 Ultraviolet Photodissociation (UVPD)

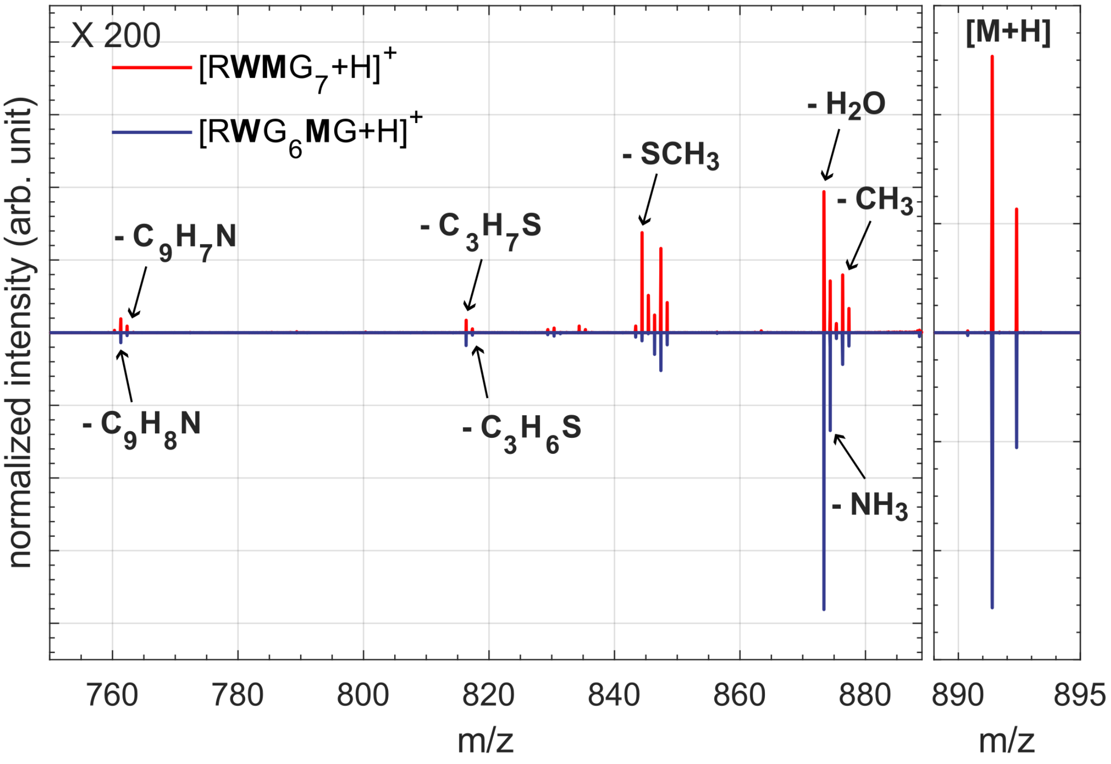

Figure S1: Photodissociation mass spectra of $[RWMG_7 + H]^+$ (red line) and $[RWG_6MG + H]^+$ (blue line, sign-inverted intensity) measured at 266 nm.

Data Treatment. The mass-to-charge spectra of each sample were calibrated with a constant mass-to-charge shift over the entire spectrum using the well-known parent mass. This shift was in all cases smaller than 0.01 *m/z*. The mass spectra were normalized to the parent peak intensity (observe the equal contribution of the four samples of $[M+H]^+$ in Figure 1 in the main article, and Figure S1 in SI). The identified fragment peaks in each mass spectrum were least-squares fitted using a Gaussian function to differentiate between overlapping fragments. To account for differences in the overall signal intensity between the four samples and obtain the fragment's relative contribution, the intensities of each fragment were summed across all samples. The intensity of that fragment in each individual sample was then normalized by dividing by this sum. The resulting relative contributions (relative branching ratio) are presented in a stacked bar plot.

1.2 Near-Edge X-ray Absorption Mass Spectrometry (NEXAMS)

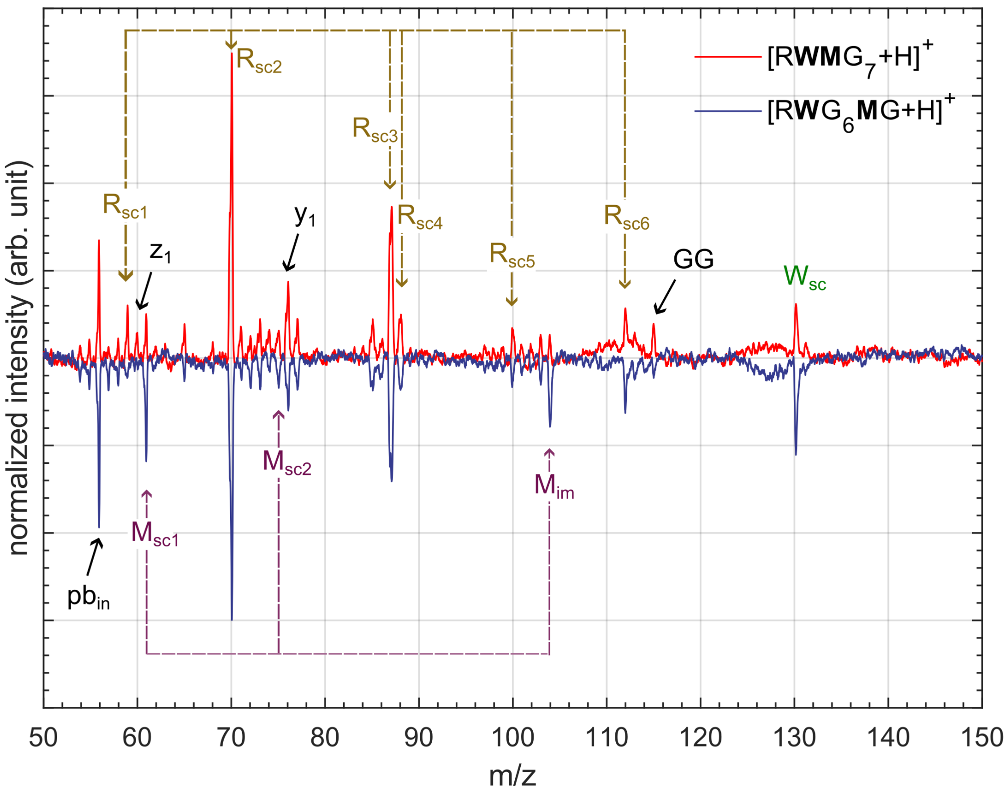

Figure S2: Photodissociation mass spectra of $[RWMG_7 + H]^+$ (red line) and $[RWG_6MG + H]^+$ (blue line, sign-inverted intensity) measured at 288 eV. sc: side-chain fragment, im: immonium ion.

Data Treatment. The data processing involved subtracting the background signal from the measured data to account for fragment ions generated by buffer gas collisions. For each photon energy, the corresponding mass spectrum was analyzed by integrating the intensity of each mass peak. Normalization on the precursor ion was not feasible due to the limited *m/z* window. The resulting yield was then normalized to the measured photon flux at each photon energy, leading to the partial ion yield spectra for each fragment. Summing the partial ion yield of all fragments across the measured spectra results in the total ion yield spectrum. To obtain the fragment's relative contribution, the intensities of each fragment were summed across all samples. The intensity of that fragment in each individual sample was then normalized by dividing by this sum. The resulting relative contributions (relative branching ratio) are presented in a stacked bar plot.

Table S1: Combined list of observed fragments for $[RWMG_7 + H]^+$ from NEXAMS, UVPD, CID (multiple collision), and CID (single collision) experiments. sc: side-chain fragment, im: immonium ion, pb_{in}: internal peptid bond fragment.

m/z	Assignment	Method	Reference
56.0	pb_{in}	NEXAMS	[1,2]
59.0	R_{sc1}	NEXAMS	[3]
60.0	z_1	NEXAMS	-
61.0	M_{sc1}	NEXAMS	[1,4]
70.1	R_{sc2}	NEXAMS, CID (multiple collision)	[1,4]
75.0	M_{sc2}	NEXAMS	[5]
76.0	y_1	NEXAMS, CID (multiple collision)	-
87.1	R_{sc3}	NEXAMS, CID (multiple collision)	[4]
88.0	R_{sc4}	NEXAMS	[1,4]
100.1	R_{sc5}	NEXAMS, CID (multiple collision)	[4]
104.1	M_{im}	NEXAMS, CID (multiple collision)	[1,4]
112.1	R_{sc6}	NEXAMS, CID (multiple collision)	[1,4]
115.1	GG	NEXAMS, CID (multiple collision)	[2]
117.0	z_2	NEXAMS, CID (multiple collision)	-
130.5	W_{sc}	NEXAMS, CID (multiple collision)	[1,4]
133.1	y_2	CID (multiple collision)	-
144.1	GGG-CO	CID (multiple collision)	-
159.1	W_{im}	CID (multiple collision)	-
161.1	MG-CO	CID (multiple collision)	-
172.1	GGG	CID (multiple collision)	-
183.9	MGG-C_2H_6S	CID (multiple collision)	-
229.1	GGGG	CID (multiple collision)	-
298.2	a_2-NH_3	CID (multiple collision)	-
315.2	a_2	UVPD	-
326.2	b_2-NH_3	CID (multiple collision)	-
361.1	y_6	CID (single collision)	-
503.3	a_4	CID (single collision)	-
588.3	b_5	CID (single collision)	-

m/z	Assignment	Method	Reference
600.3	a_6-NH_3	CID (single collision)	-
617.3	a_6	CID (single collision)	-
628.3	b_6-NH_3	CID (single collision)	-
645.3	b_6	CID (single collision)	-
674.3	a_7	CID (single collision)	-
685.3	b_7-NH_3	CID (single collision)	-
735.3	y_9	CID (single collision)	-
742.3	b_8-NH_3	CID (single collision)	-
761.3	[M+H - C_9H_8N]	UVPD	[6]
762.3	[M+H - C_9H_7N]	UVPD	[6]
771.3	a_9-NH_3	CID (single collision)	-
788.4	a_9	CID (single collision)	-
816.4	[M+H - C_3H_7S]	UVPD, CID (single collision)	[6]
817.4	[M+H - C_3H_6S]	UVPD, CID (single collision)	-
834.4	b_9+H_2O	CID (single collision)	-
844.4	[M+H - SCH_3]	UVPD	[6]
873.4	[M+H - H_2O]	UVPD, CID (single collision)	-
874.4	[M+H - NH_3]	UVPD, CID (single collision)	-
876.4	[M+H - CH_3]	UVPD	[6]
891.4	$[M+H]^+$	CID (multiple collision), CID (single collision), UVPD	

2 Computational Methods

Sampling of the $[RWMG_7{+}H]^+$ peptide conformational space was performed using temperature replica exchange molecular dynamics (REMD)[7] method within the CHARMM36 force field[8] in the GROMACS software.[9] Prior to the replica exchange simulation, the $[RWMG_7 + H]^+$ structure was refined by energy minimization using molecular dynamics (MD) subjected to steepest-descent reaching a force convergence. The exchange involved nine replicas ranging 270 to 620 K, having an average desired exchange probability (P_{des}) of $\sim$0.3. These temperatures—270, 302, 337, 375, 417, 462, 512, 567, and 620 K—were selected using a temperature predictor.[10] Each replica was simulated in gas phase with a 2 fs MD time step for 40 ns, constraining bonds involving hydrogen using the LINCS algorithm[11] and modified Berendsen thermostat.[12] All interactions such as electrostatics and van der Waals were calculated up to a cutoff of 6.0 nm – ensuring that all atoms within a molecule can interact with all other atoms of that same molecule at all times.

All the electronic structure calculations were performed using the ORCA program package.[13] The selected structures from the trajectory at 302 K were optimized prior to the excited states and the X-ray absorption spectra calculation using the Density functional theory (DFT) in combination with the restricted-open-shell configuration interaction with singles excitations (ROCIS)[14], with the B3LYP/zora-TZVP functional[15] considering relativistic and spin-orbit corrections.[16] The excited states were calculated on the active space of the core molecular orbitals, exclusively of all carbon atoms, and the corresponding C K-edge in the X-ray absorption spectra. The transition dipole moment operator combined the electric dipole, magnetic dipole, and the electric quadrupole for the spectra calculation. An atomic contribution electron transition density analysis[17] was performed over the calculated excited states and spectra.

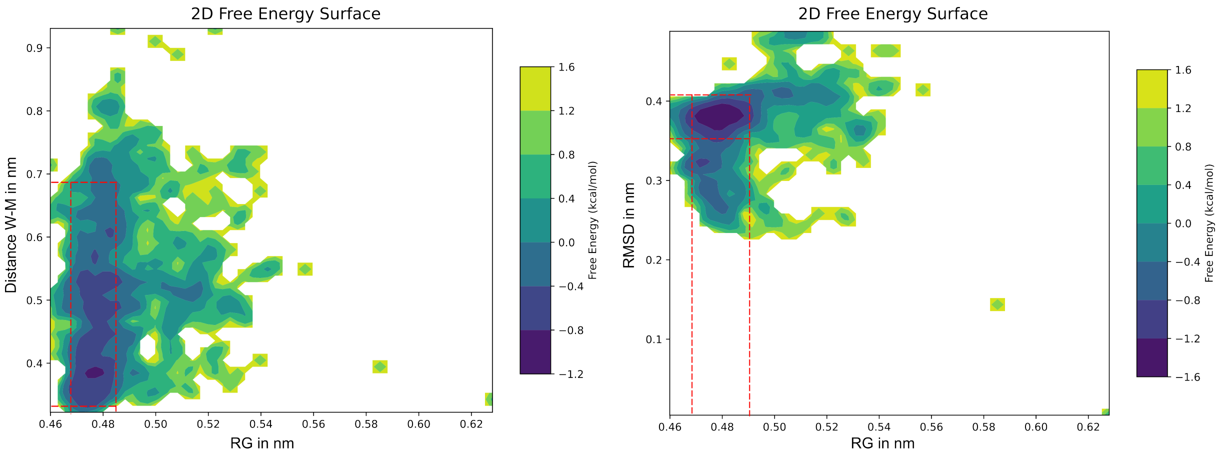

Figure S3: Potential energy surface analysis for structure selection following GROMACS simulation. Left: Radius of gyration (Rg) plotted against the distance between methionine and tryptophan side chains. Right: Radius of gyration (Rg) plotted against the root mean square deviation (RMSD).The red area in each plot indicates the region used for structure selection.

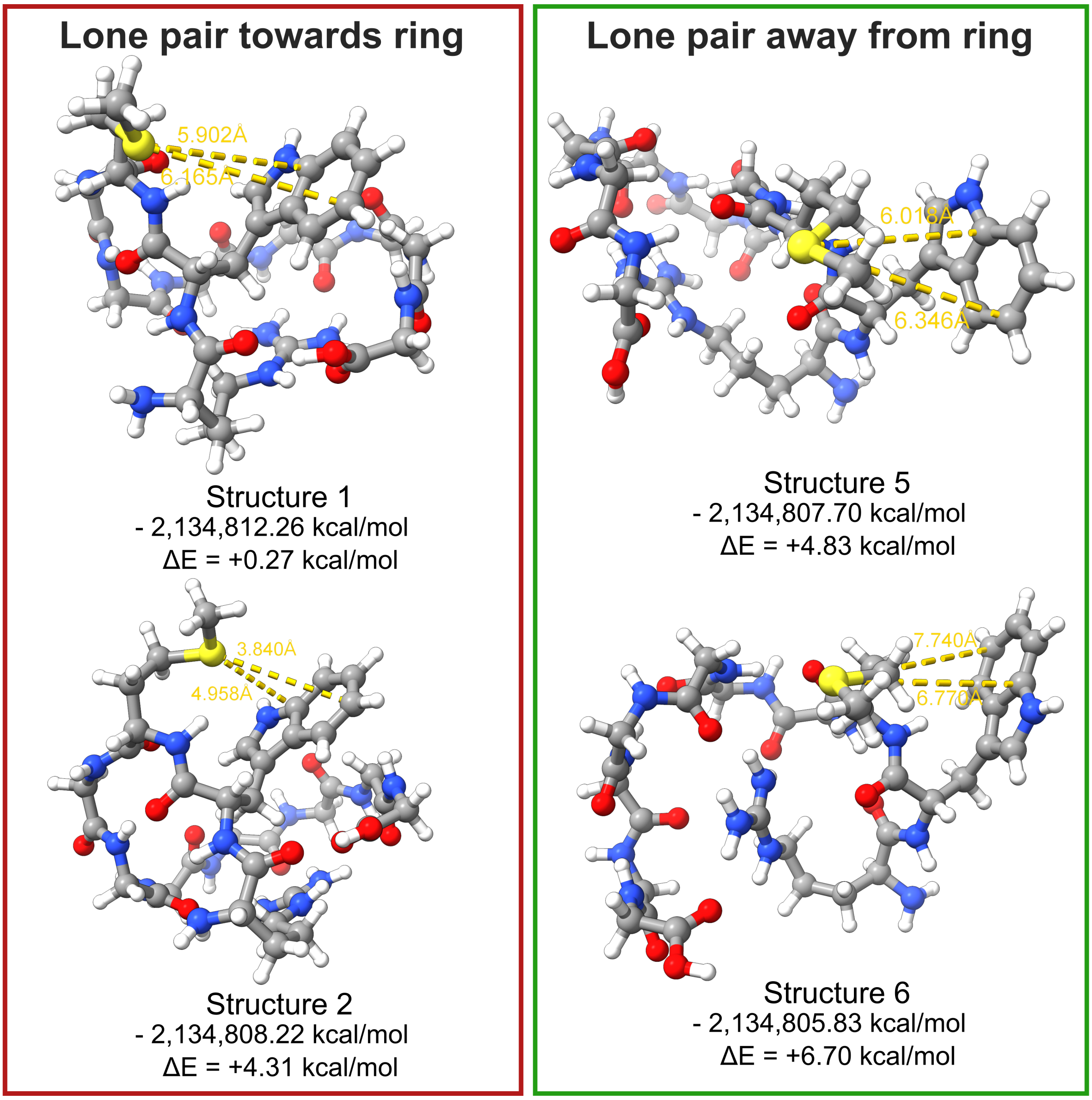

Figure S4: Overview of four structures obtained from REMD simulations (GROMACS, CHARMM36) and DFT optimization (ORCA, B3LYP/zora-TZVP). Left: Structures with the lone pair pointing towards the ring. Right: Structures with the lone pair pointing away from the ring.

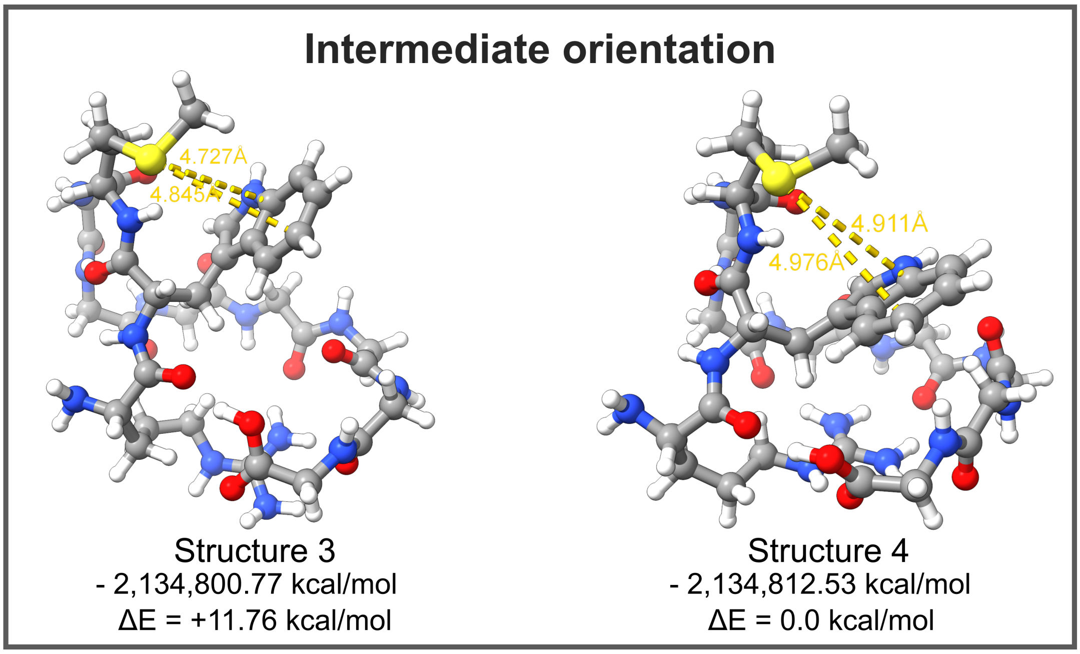

Figure S5: Overview of two structures with an intermediate lone pair orientation obtained from REMD simulations (GROMACS, CHARMM36) and DFT optimization (ORCA, B3LYP/zora-TZVP).

References

[1] Zhang, P.; Chan, W.; Ang, I. L.; Wei, R.; Lam, M. M. T.; Lei, K. M. K.; Poon, T. C. W. Revisiting fragmentation reactions of protonated α-amino acids by high-resolution electrospray ionization tandem mass spectrometry with collision-induced dissociation. *Scientific Reports* **2019**, *9*, 6453.

[2] Leroux, J. et al. Mapping the electronic transitions of protonation sites in peptides using soft X-ray action spectroscopy. *Phys. Chem. Chem. Phys.* **2023**, *25*, 25603–25618.

[3] Falick, A. M.; Hines, W. M.; Medzihradszky, K. F.; Baldwin, M. A.; Gibson, B. W. Low-mass ions produced from peptides by high-energy collision-induced dissociation in tandem mass spectrometry. *J. Am. Soc. Mass Spectrom.* **1993**, *4*, 882–893.

[4] Papayannopoulos, I. A. The interpretation of collision-induced dissociation tandem mass spectra of peptides. *Mass Spectrom. Rev.* **1995**, *14*, 49–73.

[5] Dörner, S.; Schwob, L.; Atak, K.; Schubert, K.; Boll, R.; Schlathölter, T.; Timm, M.; Bülow, C.; Zamudio-Bayer, V.; von Issendorff, B.; Lau, J. T.; Techert, S.; Bari, S. Probing Structural Information of Gas-Phase Peptides by Near-Edge X-ray Absorption Mass Spectrometry. *J. Am. Soc. Mass Spectrom.* **2021**, *32*, 670–684.

[6] Talbert, L. E.; Julian, R. R. Methionine and Selenomethionine as Energy Transfer Acceptors for Biomolecular Structure Elucidation in the Gas Phase. *J. Am. Soc. Mass Spectrom.* **2019**, *30*, 1601–1608.

[7] Sugita, Y.; Okamoto, Y. Replica-exchange molecular dynamics method for protein folding. *Chem. Phys. Lett.* **1999**, *314*, 141–151.

[8] Yu, Y.; Kramer, A.; Venable, R. M.; Brooks, B. R.; Klauda, J. B.; Pastor, R. W. CHARMM36 lipid force field with explicit treatment of long-range dispersion: parametrization and validation for phosphatidylethanolamine, phosphatidylglycerol, and ether lipids. *J. Chem. Theory Comput.* **2021**, *17*, 1581–1595.

[9] Abraham, M. J.; Murtola, T.; Schulz, R.; Páll, S.; Smith, J. C.; Hess, B.; Lindahl, E. GROMACS: High performance molecular simulations through multi-level parallelism from laptops to supercomputers. *SoftwareX* **2015**, *1-2*, 19–25.

[10] Patriksson, A.; Van Der Spoel, D. A temperature predictor for parallel tempering simulations. *Phys. Chem. Chem. Phys.* **2008**, *10*, 2073–2077.

[11] Hess, B. P-LINCS: A parallel linear constraint solver for molecular simulation. *J. Chem. Theory Comput.* **2008**, *4*, 116–122.

[12] Bussi, G.; Donadio, D.; Parrinello, M. Canonical sampling through velocity rescaling. *The Journal of chemical physics* **2007**, *126*.

[13] Neese, F. The ORCA program system. *WIREs Comput Mol Sci* **2012**, *2*, 73–78.

[14] Roemelt, M.; Neese, F. Excited states of large open-shell molecules: An efficient, general, and spin-adapted approach based on a restricted open-shell ground state wave function. *J. Phys. Chem. A* **2013**, *117*, 3069–3083.

[15] Becke, A. D. Density-functional thermochemistry. III. The role of exact exchange. *J. Chem. Phys.* **1993**, *98*, 5648–5652.

[16] Van Lenthe, E. v.; Snijders, J.; Baerends, E. The zero order regular approximation for relativistic effects: the effect of spin-orbit coupling in closed shell molecules. *J. Chem. Phys.* **1996**, *105*, 6505–6516.

[17] Ortiz-Mahecha, C.; Schwob, L.; Leroux, J.; Bari, S.; Meißner, R. H.; Bande, A. X-ray absorption spectroscopy reveals charge transfer in π-stacked aromatic amino acids. *Physical Chemistry Chemical Physics* **2025**, *27*, 8202–8211.
